# Relationship between blood urea, protein, creatinine, triglycerides and macro-mineral concentrations with the quality and quantity of milk in dairy Holstein cows

**Published:** 2012

**Authors:** Shahram Nozad, Ali-Gholi Ramin, Gholamali Moghadam, Siamak Asri-Rezaei, Azadeh Babapour, Sina Ramin

**Affiliations:** 1 *Department of Clinical Science, Faculty of Veterinary Medicine, Urmia University, Urmia, Iran; *; 2 *Department of Clinical Science, Faculty of Veterinary Medicine, Tabriz University, Tabriz, Iran;*; 3 *Veterinary Student, Faculty of Veterinary Medicine, Urmia University;*; 4 *Medical Student, Tabriz University of Medical Sciences, Tabriz, Iran.*

**Keywords:** Holstein, Serum, Blood, Macro-minerals, Milk yield

## Abstract

Seventy six high and low producer cows were selected to determine the composition of the blood and milk parameters, and their interrelationships to determine the indices which could be useful to improve the milk yield. The highest mean blood concentrations were found in high producer cows. Mean values for blood urea nitrogen (BUN), serum protein (SPtn), creatinine, triglycerides (TGs), cholesterol, and beta-hydroxybutyric acid (BHB) were 25.10 mg dL^-1^, 10.15 g dL^-1^, 0.81, 62.30, 177.10 and 0.16 mmol L^-1^, and for macro-minerals including SCa, SMg, serum in-organic phosphorus (SIP), SNa and SK were 3.85, 2.66, 4.63, 108.00 and 4.34 mmol L^-1^, respectively. The highest concentrations for milk parameters, were observed in the high producers, and were significant only for MCa, MIP and MMg. Mean values for milk urea nitrogen (MUN), milk protein (MPtn) and lactose were 19.90 mg dL^-1^, 0.39 g dL^-1^, and 4.12% and for macro-minerals, 13.24, 3.88, 11.03, 73.30 and 16.90 mmol L^-1^, respectively. There were significant positive correlations between the blood and milk parameters except for creatinine/BHB, TGs/cholesterol and MNa/MK which were not significant. The correlations between the blood parameters were greater than in the milk parameters. Creatinine and SPtn, MUN and MPtn were the main parameters in that the relationships between MPtn with BUN, SPtn and creatinine were more noticeable than others. The regression analysis showed that BUN with the SIP and creatinine, MPtn with the BUN and creatinine and MUN with the SIP and SMg were the appropriate parameters in improvement studies related to the milk yield. In conclusion, BUN, SPtn, MUN and MPtn concentrations are the most effective indices for predicting the preferred milk yield.

## Introduction

Evaluation of the blood and milk biochemical parameters to assess the animal health and milk yield has always been stressed by authors and the various discrepancies have been observed in both blood and milk yield results.^1^^,^^2^ Milk parameters originate from blood and food components^3^ and clarifying the appropriate relationships among these parameters individually in food,^4^ blood,^5^ and milk^6^ are useful in understanding the health and production status in animals. Among blood biochemical parameters, the metabolic profile test is known as an applicable approach in assessing glucose, protein, BUN, TGs, cholesterol BHB and macro-minerals concentrations. The same tests have been adopted for the milk parameters including protein, lactose and macro-minerals as the major animal products, resulting in improved animal growth and skeletal tissues in animals.^3^^,^^5^ Milk contains 4% protein which is easily digested and its nitrogen is converted to urea in liver and excreted from blood to urine, feces, milk and other body excretions.^1^^,^^7^ Thus, MUN as an indirect and compulsory composition in the milk must also be evaluated. The normal value for MUN in cows has been reported^8^^,^^9^ up to 40 mg dL^-1 ^influenced mainly by dietary energy and protein,^10^ lactation period,^4^ and pregnancy and parturition.^11^

Cow's milk contains 0.09% dry matter minerals in which over 80% of macro-minerals are soluble in milk serum and are possible to assess, but remaining 20% is conjugated with milk casein and is not measureable in the laboratory evaluation. The concentration of Mg in milk was lower than minerals and the reported values for Ca, IP, Na, K and Mg were 48.80, 30.40, 10.80, 58.50, and 5.42 mmol L^-1^.^3^^,^^5^^,^^8^ These values were influenced by lactation period, milk yield, milk urea and protein.^12^^,^^13^ The interrelationships between macro-minerals in the blood serum, the musculoskeletal system, CSF and urine have been confirmed and published elsewhere,^14^ however this has not been the case for milk itself and between milk and blood as well. Reportedly, the correlations between milk Ca, K and Na in high producer cows, but not their relationship with blood macro-minerals.^14^

Recent reports by Nozad *et al.*^1^ on cows' MUN and MPtn together with the other findings in ruminants' MUN,^8^ MUN and food composition,^15^^,^^16^ MUN and milk quality and quantity^17^ and, finally, MUN and reproduction^18^ have provided vast information on the evaluation of milk production without reference to the importance of blood profile test and their effects on milk production. Nowadays, milk testing and permanent recording programs are run constantly on cows for dairy herd improvement and, in the case of approved relationships between milk parameters and the blood profile test, it provides an opportunity for dairy managers to organize their recording system on milk sample tests instead of blood samples, in that it is much easier, practicable and less costly than blood sampling.^6^ To achieve this goal, it is necessary to acquire the indices among blood and milk parameters such as urea and protein which could predict the real blood profile or milk quality and quantity.^19^ Thus, the aims were: 

1- Determination of the concentrations of blood and milk parameters and their comparison with the high and low producer dairy Holstein cows. 

2- Estimation of the correlations among blood, milk, and blood and milk parameters. 3- To present the appropriate indices among blood and milk parameters to improve the dairy herds productions. 

## Materials and Methods

Seventy six dairy Holstein milking cows, including 40 cows with milk yield up to 40 kg per day (high producer) and 36 with milk yield up to 20 kg per day (low producer) were selected in 2010 in Urmia (the northwest of Iran). The average daily milk production was between 29 and 32 kg per day. The high producer cows were all in the first half of lactation, while the lower producers were in the second half of lactation or they had low milk yield. Ten mL milk from the morning milking as well as 5 mL blood from the jugular vein was collected from the cows. During sampling, the individual information including the cow's ear ID number, daily milk yield, age, pregnancy and the number of parity were also obtained. The cows were fed *ad libitum* diet containing lucerne, pulp, concentrate and silage. The blood and milk samples were cooled at 4 °C in a refrigerator for 24 hours to separate blood serum and milk fat. The samples were tested for the concerned parameters (see below). During the study mastitis and other clinical diseases were monitored and the cows were all found to be in good health.

Blood sera were separated by centrifugation of samples in 3000*g* for 15 minutes. The concentrations of BUN, MPtn, creatinine, TGs, cholesterol, BHB, SCa, SMg, and SIP were assessed in an auto-analyzer machine (RA-1000, USA), BHB (Runbut, Diamond, UK) by Ultra-violet method in spectrophotometer, and SNa and SK in a flame photometer (Jenway, Clinical PFP7, UK) using an appropriate kit for each measurement (Pars Azmon, Iran, Rumbut, UK). Results were calculated in standard units. Milk samples were first defatted by placing them in a cool area (4 °C) and centrifugation in 3000*g* for 5 minutes. Milk casein was separated by 0.1 N HCl in pH 3.6. Milk sera were used to determine the MUN, MPtn and macro-mineral concentrations. MUN and MPtn were measured the same as for BUN and SPtn. Whole milk was used to determine the milk lactose and was evaluated by Teles method and lactose standard in a spectrophotometer. Milk Calcium, MIP, MMg, MNa and MK concentrations were assessed as for blood minerals in an auto-analyzer and a flame photometer machine. 

Data were analyzed by SPSS (version 13) and mean ± SEM were determined for all parameters in the blood and milk samples. Student *t*-test was carried out to reveal the differences among the parameters. Pearson correlation test was used to evaluate the relationships among parameters in different milking yields. Regression test was applied to make up the suitable equations among blood, milk and between blood and milk parameters.

## Results

The means for BUN, SPtn and macro-mineral concentrations in high producer cows were significantly (*P*<0.05) greater and cholesterol was lower than in low producer cows (Table 1). The mean concentrations for BUN, SPtn, creatinine, TGs, cholesterol, BHB, SCa, SMg, SIP, SNa and SK were within the normal range. The highest blood parameter concentrations with the exception of cholesterol were observed in high producer cows. The means for milk parameters excluding lactose and MIP in high producers were greater than in low producer cows, in which it was significant (*P* < 0.05) only for MCa, MMg and MIP (Table 2). The mean concentrations for MUN, MPtn, lactose, MCa, MMg, MIP, MNa and MK were within the normal range. Comparison of corresponding parameters with another one between the blood and milk demonstrated a significant difference (*P* < 0.05).

**Table 1:** Mean ± SE blood parameters in high and low producer dairy Holstein cows (n=76).

**Parameters**	**High producer cows**	**Low producer cows**	**Overall**
**Urea** 1	26.50±0.78 a	23.60±0.65 b	25.13±0.53 a
**Protein** 1	10.40±0.21 a	9.90±0.16 b	10.15±0.14 a
**Creatinine** 1	0.86±0.02 a	0.75±0.02 b	0.81±0.02 a
**Triglyceride** 1	27.5±2.80 a	25.10±1.20 a	26.30±1.60 a
**Cholestrol** 1	162.6±6.80 a	192.90±8.03 b	177.10±5.50 a
**BHB** 2	0.15±0.01 a	0.17±0.07 a	0.16±0.01 a
**Calcium** 2	1.83±0.05 a	1.60±0.04 b	1.71±0.03 a
**Magnesium** 2	1.17±0.06 a	1.01±0.05 b	1.09±0.04 a
**Phosphorus** 2	1.62±0.05 a	1.35±0.04 b	1.50±0.04 a
**Sodium** 2	108.6±0.7 a	107.40±0.95 a	108±0.58 a
**Potassium** 2	4.50±0.07 a	4.29±0.07 a	4.34±0.05 a

a,b Different letters in each row differed significantly (*P* < 0.05).

1 indicates mg dL^-1^,

2 indicates mmol L^-1^.

**Table 2:** Mean ± SE milk parameters in high and low producer dairy Holstein cows (n=76).

**Parameters**	**High producer cows**	**Low producer cows**	**Overall**
**Urea** 1	20.10±0.42 a	19.70±0.31^a^	19.90±0.26
**Protein** 1	0.40±0.00 a	0.38±0.00 a	0.39±0.00
**Lactose** 1	4.11±0.04 a	4.13±0.04 a	4.12±0.02
**Calcium** 2	13.41±0.95 a	13.08±0.13 b	13.24±0.07
**Magnesium** 3	3.98±0.05 a	3.78±0.04 b	3.88±0.03
**Phosphorus** 3	10.90±0.17 a	11.04±0.16 a	11.03±0.12
**Sodium** 3	76.60±2.10 a	69.40±2.04 b	73.30±1.50
**Potassium** 3	16.90±0.29 a	16.80±0.34 a	16.90±0.22

a,b Different letters in each row differed significantly (*P* < 0.05).

1 indicates mg dL^-1^,

2 indicates percentage (%),

3 indicates mmol L^-1^.

The results of the correlations among blood (Table 3), milk (Table 4), and blood and milk parameters (Table 5) showed significant negative relationships (*P* < 0.01) between creatinine/BHB, TGs/cholesterol and MNa/MK, and positive correlations among other parameters. The highest correlations were observed between blood parameters (n = 15) and the lowest between milk parameters (n = 6) and for mixed blood and milk parameters, 8 cases (Table 3). Creatinine, MUN and MPtn were the main parameters in that the relationships between MPtn with BUN, SPtn and creatinine were more noticeable than others (Table 5). The regression results showed equations among BUN, BIP, creatinine (R^2 ^= 0.23, SE = 3.38) and milk protein, creatinine, MUN (R^2 ^= 0.19, SE = 0.05) and MUN, SIP, SMg (R^2 ^= 0.17, SE = 0.79). The equations were as mentioned, and are listed below:

*BUN (mg dL* ^-1^ *) = 9.45 -* *]* *(10.3* *±* *3.5) Creat +* *(1.6 ± 0.52) SIP], R*^2 ^*= 0.23, SE = 3.38*

*Milk Protein (mg dL* ^-1^ *) = 0.2 -* *]* *(0.13* *±* *0.4)Creat + (0.14* *±* *0* *.07)MUN], R* ^2^ *= 0.19, SE = 0.05*

*MUN* *(mg dL*^-1^*) = 4.19 - ](0.16 ± 0.7)* *SIP + (0.32 ±* *0.1) SMg]*

*R* ^2^ *= 0.17, SE = 0.79*

**Table 3:** Correlations among blood parameters in dairy Holstein cows (n=76).

**Parameters**	**Protein**	**Creatinine**	**BHB**	**Cholesterol**	**Ca**	**Mg**	**IP**	**K**
**Urea**	*0.34	*0.36	-	-	-	-	*0.37	-
**Protein**	-	-	-	-	*0.25	*0.36	*0.52	-
**Creatinine**	*0.35	-	*0.23	-	*0.23	*0.26	-	-
**Triglycerides**	-	-	-	*0.24	-	-	-	*0.28
**Calcium**	*0.35	*0.23	-	-	-	-	*0.31	-

Blood Na showed no correlation with other blood parameters.

* indicates *P* < 0.01.

**Table 4:** Correlations among milk parameters in dairy Holstein cows (n=76).

**Parameters**	**Protein**	**IP**	**Na**	**K**
**Urea**	*0.24	-	-	**0.62
**Lactose**	-	**0.34	**0.76	-
**Mg**	-	-	*0.28	-
**Na**	-	-	-	**0.74

Milk Ca and Mg showed no correlations with other milk parameters.

* indicates *P* < 0.05,

** indicates *P* < 0.01.

**Table 5:** Correlations among blood and milk parameters in dairy Holstein cows (n=76).

**Parameters**	**MUN**	**Milk Protein**	**Milk Mg**	**Milk Na**	**Milk K**
**Blood Urea**	-	*0.25	-	-	**0.29
**Blood Protein**	*0.24	**0.23	-	-	-
**Creatinine**	-	*0.25	*0.23	-	-
**Triglycerides**	-	-	-	*0.26	-
**Blood Mg**	*0.25	-	-	-	-

* indicates *P* < 0.05,

** indicates *P* < 0.01.

## Discussion

Evaluation of the BUN, SPtn, TGs, cholesterol, creatinine, BHB and macro-minerals provides an opportunity to expect the healthy production in animals. This is known as blood profile test. The result of this study reveals an optimal condition for production in dairy cows. BUN, SPtn and BHB have been widely used in animals to demonstrate the level of energy and protein necessary for production. The low level of BUN in comparison with other findings,^2^^,^^4^ the normal range of SPtn and low amount of BHB, all together show the conditions for optimal production. BUN might increase following water deprivation, thirst, diarrhea,^20^ urinary diseases,^3^ pregnancy toxemia^9^ and acidosis,^21^ which were not case for these cows. However, individual differences in serum parameter concentrations among cows indicate that the concentrations among cows appear to be varied from one cow to another, as they are also reflective of the body metabolism, amount of milk yield and the level of food consumption. Although the differences were statistically significant, as they were within normal range they were not biologically important. The lowest concentration was found for BHB and indicated an appropriate fat metabolism in the presence of glucose and oxaloacetate, and this interpretation was supported by the level of TGs and cholesterol observed for these cows.^2^

Serum macro-mineral concentrations were in better agreement with Gibasiewicz's report^22^ and disagreed with Jacob *et al.*’s findings,^23^ but in normal range as described for cows.^3^ The slight discrepancies could be related to the animal breeding system and nutritional management.^24^ The role of SCa and SMg are much more visible than other minerals and their balances in the body resulted in improvements in production, reproduction, and growth performance, and as well as improvement in the health status of animals. Disorders following mineral deficiencies are more common than a high level of concentration in the serum.^3^ Hypomagnesaemia itself increases the risk of milk fever and milk titan.^25^ Hypocalcemia retards food intake, and therefore, reduces milk yield and increases Vagus indigestion and diarrhea.^3^^,^^26^ Hyponatremia is not common in cattle and hypophosphatemia causes pica and post parturient hypoglobinemia.^3^ The observation of mineral concentrations within normal range indicates sufficient mineral supplementation in their diet. The individual differences among macro-mineral concentrations could be related to the level of daily food intake,^2^ the amount of milk yield and pregnancy status.^23^

The mean MUN and MPtn were slightly lower than other reports, but were in agreement with the authors regarding lactose.^27^ Milk protein is considered to be one of the main ingredients in milk price, thus it has a direct effect on milk quality. MUN is the result of the protein metabolism in the liver and these parameters are directly related to the blood and diet composition. The recommended values for high and low producer cows were 16% and 14%, respectively.^2^ The low level of MUN observed in this study indicates an appropriate condition for dairy milk production, but it could also be an indication of low protein consumption in the diet as reported by some authors.^10^ Variations in MUN, MPtn and lactose were shown during milking period,^4^ the amount of milk yield^8^ and pregnancy.^27^ Milk macro-mineral concentrations in cows, except for MNa, were a few folds of that in the blood but less than other findings in milk.^2^ This means that these concentrations are not stable in the milk composition and could be influenced by the level of dietary protein, MUN and milk production.^28^ Reportedly, MCa is higher in high producer cows than in low producers, which is not in agreement with this study.^14^ The amount of MCa absorption from the intestines depends directly on MMg. Although the amount of MCa is 10 times that of MMg, the absorption rate for both minerals from the intestines is the same. This means that the huge amounts of MCa are not absorbed due to low MMg. The lack of MCa and MIP causes malformation of the skeletal system and rickets,^3^ while a high level of these minerals in the milk of healthy cows is an appropriate condition in milk production. This condition can appear at the beginning and the end of lactation periods.^8^ The MMg in high producer cows, even in hypomagnesaemia and diet with low Mg is constant and is from 3 to 4 g per day depending on the level of Mg in food, milk yields and disease outbreaks. Low MMg was reported normally in high producer cows,^14^ while high MMg induces milk fever in cows and improves tetany in calves.^20^ The high MMg in low producer cows could be related to the high density and low volume of milk.^9^ The differences in the MNa and MK between the both producer cows also show that these variations involved all macro-minerals including MNa and MK,^15^ but the importance of MCa and MMg is more apparent than other minerals, and although no reports were found to show disorders following low MK or low absorption from the intestines, MK and salts increase following mastitis, while MNa drops during estrus.^19^ The relationships observed between the two samples in this study reveal that some parameters could be useful and valuable indices to predict the health status, production and reproduction conditions of cows. The cooperation of indices in blood parameters was greater than in milk. The main indices are SPtn, BUN, creatinine, MPtn, MUN and MNa.

Milk Ca and SNa revealed no correlations with the other parameters, while creatinine, one of the health indexes, and MPtn, had the greatest correlations with others. Among minerals, the correlations between Na/K and Ca/IP were visible as mentioned by others.^20^^,^^23^ The cooperation of milk minerals in the formation of teeth and bones, blood osmolarity balances, acid-base status and muscular contraction^3^ accompanied by other reports among BUN, BHB and SPtn in blood,^22^ MUN, MPtn and lactose^1^ in milk and MPtn, SPtn, BUN, and creatinine in this study confirm all previous^4^^,^^20^^,^^29^^,^^30^ and recent findings that the correlations between these parameters could be used in improvement of animal production and reproduction performances. 

The result of the correlations and regressions reveal that BUN, creatinine, SIP and MPtn, MUN, creatinine and MUN, MPtn, and SMg are suitable equations for animal breeding programs. Although the R^2^ were slightly low, they seem appropriate for future studies in which, by adding the blood glucose and increasing the number of experimental animals, it might be possible to achieve acceptable results.^2^ Protein and urea in both samples are suitable for the mentioned studies, while creatinine is not suitable to make the equation. This was in agreement with other findings that MUN has the capacity to predict the dietary nutrients of animals, production and reproduction performances as well.^1^^,^^2^^,^^29^ MUN negatively affected estrus, open days, milk composition and hygiene.^17^ It is widely used in the evaluation of the dietary protein, urine urea, ruminal ammonium,^20^^,^^23^^,^^27^ BUN and dietary energy.^4^^,^^29^

It is concluded that the concentrations of parameters in high producers were greater than in low producing ones. The relationships in the blood parameters were the highest and in milk were the lowest. Creatinine, milk and serum protein and MNa have the highest correlations with the other parameters. The relationships among milk protein, serum protein, BUN and creatinine were more visible than others and, finally, serum parameters except for protein, creatinine and SMg were not suitable to predict the quality and quantity of cows' milk.
